# Pygo1 Regulates the Behavior of Human Non-Small-Cell Lung Cancer via the Wnt/*β*-Catenin Pathway

**DOI:** 10.1155/2022/6993994

**Published:** 2022-11-08

**Authors:** Yao Wen, Yuling Li, Boyu Yang, Xiangrong Guo, Li Lin, Jifeng Liang, Kai Zhang, Xu Li, Zhigang Jiang, Yuequn Wang, Yongqing Li, Xiushan Wu, Xiongwei Fan, Fang Li, Wuzhou Yuan

**Affiliations:** ^1^State Key Laboratory of Development Biology of Freshwater Fish, The Center for Heart Development, The College of Life Sciences, Hunan Normal University, Changsha, Hunan 410081, China; ^2^National Clinical Research Center for Geriatric Disorders, Department of Geriatrics, Xiangya Hospital, Central South University, Changsha, Hunan 410008, China; ^3^The Second Department of Thoracic Surgery, Hunan Cancer Hospital and the Affiliated Cancer Hospital of Xiangya School of Medicine, Central South University, Changsha, Hunan 410013, China; ^4^Guangdong Provincial Key Laboratory of Pathogenesis, Targeted Prevention and Treatment of Heart Disease, Guangzhou, Guangdong 510000, China

## Abstract

Abnormal activation of the classical Wnt pathway has been reported in non-small-cell lung cancer (NSCLC) previously. Pygo family genes, the core regulators of Wnt/*β*-catenin signaling, were also reported to be involved in tumorigenesis. However, the role of the homolog Pygo1 in human lung cancer remains unclear. In the current study, we demonstrated an association of increased Pygo1 expression with consistent high nuclear *β*-catenin signals across pathological tissue samples of early-stage human NSCLC. Overexpression of Pygo1 in lung cancer cells resulted in enhanced G1/S cell phase transformation, reduced apoptosis, and increased cell proliferation. These changes were accompanied by the downregulation of cell cycle-related proteins, such as RB, p16, p53, and p27Kip1, and increased expression of CyclinE1. Migration, wound healing, and colony formation assays revealed that Pygo1 overexpression enhanced the invasion and migration of lung cancer cells, increased the formation of clones, and suppressed E-cadherin expression. In addition, overexpression of Pygo1 in lung cancer cells led to an increase of *β*-catenin/TCF4 complex, as well as upregulated expression of target genes of *β*-catenin. *In vivo* experiments also revealed that Pygo1 overexpression promoted the tumorigenicity of a xenograft tumor model, while Wnt inhibition partially blocked the effect of Pygo1 overexpression. In conclusion, Pygo1 affects human NSCLC via the canonical Wnt/*β*-catenin pathway, which provides new clues for lung cancer pathology.

## 1. Introduction

Lung cancer is the main cause of cancer-related deaths worldwide [1], and the malignancy of cancer is correlated with the most rapid increases in morbidity and mortality. Human lung cancers are subdivided into small cell lung cancer and non-small-cell lung cancer (NSCLC), of which the latter accounts for approximately 85% of all cases. Lung adenocarcinoma (LUAD) and lung squamous cell carcinoma (LUSC) are the most common subtypes of NSCLC [2]. Although important advances in the current treatment of NSCLC have improved our understanding of the disease pathology and tumor progression mechanisms [3], the mortality associated with lung cancer remains high, as indicated by a 5-year relative survival rate of only 19% [4]. Therefore, our understanding of the etiology of lung cancer and the ability to promote effective treatments in the future rely on the identification of new cancer-inducing factors and inhibitors [5].

In animals, the Wnt signaling pathway is considered highly important [6], which has been shown to activate signal transduction and trigger changes in gene expression and cell behaviors such as cell adhesion and cell polarity [7]. The canonical Wnt pathways begins with the binding of the Wnt ligand to the cysteine-rich domain of the Wnt receptor, a frizzled (Fzd) family protein, on the cell membrane [7]. Wnt activity stabilizes *β*-catenin [8], which forms a destructive complex with the scaffold protein Axin and adenomatous polyposis coli in the absence of the Wnt ligand [9]. The complex phosphorylates *β*-catenin and further induces its ubiquitination and degradation to maintain a low *β*-catenin level in the cytoplasm. In the absence of nuclear *β*-catenin, histone deacetylases (HDACs) are recruited by inhibitor complexes containing T cell factor (TCF)/lymphoid enhancer-binding factor 1 (LEF) and transducing-like enhancer protein (TLE/Groucho) to prevent the expression of target genes [10]. In the presence of Wnt ligand, however, the stable *β*-catenin protein enters the nucleus and forms a complex with TCF/LEF and B cell lymphoma-9 (Bcl-9) to regulate the expression of downstream target genes [7].

Abnormal activation of the classical Wnt signaling pathway has been identified in a variety of human cancers. For example, the tumorigenesis and prognosis of NSCLC are associated with the abnormal activation of Wnt, which is accompanied by the silencing of Wnt inhibitory factor-1 and the methylation of secreted Fzd-related proteins [11]. However, most mutations in genes encoding key factors in the Wnt signaling pathway, including *β*-catenin, are associated with colon cancer or colorectal adenomatous polyposis but rare in lung cancer [7], suggesting that the Wnt signaling pathway may be activated upstream of *β*-catenin in lung cancer.

Pygopus (Pygo) has been identified as a critical component of canonical Wnt signaling in genetic *Drosophila* studies. It was reported that the nuclear accumulation of *β*-catenin required the interaction of Pygo and Lgs during the formation of a typical transcription complex [12]. It has been proposed that once tethered to *β*-catenin/TCF, the N-terminal homology domain of Pygo activates the expression of target genes [13], such as those encoding c-Myc, CyclinD1, and survivin. Previous studies have reported that the homologs Pygo1 and Pygo2 are not absolutely required for canonical Wnt signaling in most developing systems but rather function as quantitative transducers or modulators of Wnt signal intensity [13]. Pygo2 overexpression has been identified as an important contributor to abnormal Wnt activation in human lung cancer and has become a putative therapeutic target [14]. However, little is known about the role of Pygo1 in human lung cancer. Here, we attempted to explore whether Pygo1 participates in abnormal activation of Wnt signaling pathway I in human NSCLC and whether the interaction between Pygo1 and *β*-catenin affects lung cancer development.

## 2. Materials and Methods

### 2.1. Analysis of the Public Cancer Genomic Dataset

To investigate the clinical relevance of Pygo1 in NSCLC, the Kaplan-Meier plotter (http://kmplot.com/analysis/index.php?p=service&cancer=lung) was used to analyze the impact of Pygo1 on overall survival (OS) in lung cancer patients. The Gene Expression Omnibus database (GEO), Cancer Genome Atlas (TCGA), and European Genome-Phenome Archive (EGA) were reassessed. The resulting curves were plotted based on the Pygo1 expression value.

### 2.2. Clinical Specimens, Cell Culture, and Transfection

The study protocol was approved by the Institutional Ethics Committee of Hunan Normal University and Hunan Cancer Hospital. Written informed consent was obtained from each subject. Tissue samples were obtained from 70 patients with NSCLC who had undergone lung cancer resection surgery between 2018 and 2019 at Hunan Cancer Hospital. The pathological diagnoses and grades of all NSCLC samples were assigned according to the World Health Organization (WHO) classification system. Both NSCLC tumor tissues and adjacent normal tissues were removed during the resection surgeries.

A549 cells (epidermal growth factor receptor wild-type cells, WELL BIOLOGICAL SCIENCE) were cultured in RPMI-1640 medium complemented with 10% fetal bovine serum (Gibco, USA) and 100 U/ml streptomycin/penicillin (TransGen Biotech, China). Lipofectamine 2000 reagent (US Life Technologies) was used for the transient transfection of cells with a Tag2B-labeled Pygo1-encoding plasmid and corresponding empty plasmid vector, according to the manufacturer's instructions. Another group was transfected with Tag2B-labeled Pygo1-encoding plasmid and corresponding empty plasmid vector and both treated with Wnt inhibitor KYA1797K (Selleck Chemicals, S8327). Besides, the cells transfected with Tag2B-labeled Pygo1-encoding plasmid were transferred to a 6-well plate for selection with G418 (1000 *μ*g/ml; Sigma-Aldrich, USA). Stable transfectants were incubated in conventional medium with the addition of G418 (500 *μ*g/ml).

### 2.3. Immunohistochemistry

Patient samples were fixed with 4% paraformaldehyde in 0.1 M phosphate buffer, embedded in paraffin, and sliced into 4 *μ*m sections. Immunostaining was performed as previously described [15, 16]. The sections were incubated with a monoclonal anti-Pygo1 antibody (1 : 50; Abcam, ab170607) at 4°C overnight and subsequently incubated with a biotin-conjugated secondary antibody for 45 min. The sections were washed with TBST and incubated with horseradish peroxidase- (HRP-) conjugated streptavidin–biotin (ServiceBio, China). The colorimetric reactions were developed using 3,3-diaminobenzidine tetrahydrochloride (Sinopharm Chemical Reagent, China). An open-source plugin called “IHC Profiler” was used in this study. The formula for calculating score levels was as follows: high positive was 4 points, positive was 3 points, low positive was 2 points, and negative was 1 point [17].

### 2.4. Flow Cytometry

A549 cells were harvested 48 h after transfection with the Pygo1 overexpression plasmid or empty vector control plasmid. More than 10^5^ cells were trypsinized, collected in a microcentrifuge tube, resuspended, and washed 3 times with ice-cold phosphate-buffered saline (PBS). After centrifugation at 1000 × *g* for 5 min, the supernatant was discarded and the cells were fixed with 70% ice-cold ethanol overnight at 4°C prior to resuspension in 0.5 ml of PI/RNase Staining Buffer. After a 10 min incubation at room temperature in a dark room, the cell cycle phases and apoptosis were analyzed using a Gallios Flow Cytometer (Beckman, USA).

### 2.5. CCK-8 Cell Proliferation Assay

The cell proliferation rates were measured using Cell Counting Kit-8 (CCK-8; Beyotime, Hangzhou, China). A total of 5 × 10^3^ A549 cells were seeded in each well of a 96-well plate for 24 h, transfected with the indicated plasmid and/or inhibitor, and incubated for an additional 24, 48, or 72 h. 10 ml of CCK-8 reagent was added to each well 1 h before the endpoint of the incubation. Finally, the optical density at 450 nm (OD_450_) in each well was determined by a microplate reader.

### 2.6. Cell Invasion and Migration Assay

An invasion assay was conducted using Transwell inserts with 8 *μ*m pores (Corning, USA). At 48 h after transfection, 3.0 × 10^5^ A549 cells in serum-free medium were added to each Matrigel matrix-precoated (BD, USA) upper well insert. Then, 500 *μ*l of medium containing 10% fetal bovine serum was added to each lower chamber. After a 48 h incubation, the noninvaded cells were removed from the upper surfaces of the Transwell membranes with a cotton swab, and the cells that had invaded the lower membrane surface were fixed in methanol, stained with 0.5% crystal violet, photographed, and counted. In addition, the chambers were removed, placed in 500 *μ*l of 10% acetic acid, shaken slowly to decolorize the chambers, and subjected to a measurement of the absorbance (OD) value at 550 nm using an enzyme-standard instrument.

For the migration assay, the cells were digested with trypsin, counted, and seeded in a 6-well plate at a density of approximately 5 × 10^5^ cells per well. Horizontal lines were evenly drawn on the back of the orifice plate. Once the cells formed an adherent monolayer, a pipette tip and a ruler were used to draw a horizontal line perpendicular to the previous drawing. The cells were washed 3 times with sterile PBS to remove the detached cells, and the medium was replaced with serum-free Dulbecco's Modified Eagle's Medium. The scratches were imaged at baseline (0 h) and cultured at 37°C and 5% CO_2_ for 24 and 48 h. An image was collected at each time point.

### 2.7. Colony Formation Assay

After transfection and inhibitor treatment, cells were plated evenly in 60 mm culture dishes at a density of 1.2 × 10^3^ cells per dish and were incubated for 2 weeks. The formed colonies were fixed with 10% formalin, stained with Giemsa, and counted.

### 2.8. Western Blotting

The cells were harvested and washed with ice-cold PBS. After aspirating the supernatant, the cells were resuspended with ice-cold RIPA buffer containing protease and phosphatase inhibitor cocktails (Abcam, USA). The cell suspensions were continuously agitated for 30 min at 4°C and then centrifuged in a microcentrifuge at 12,000 × *g* and 4°C for 15 min. The supernatants were moved to fresh microcentrifuge tubes placed on ice. A nuclear protein extraction kit (EpiZyme, China) was used for nuclear protein extraction. Equal amounts of protein lysates were loaded onto a SDS-PAGE gel, separated by electrophoresis, and electrotransferred to nitrocellulose membranes (Bio-Rad, USA). After a 2 h incubation in 5% nonfat dried milk in TBST at room temperature to block nonspecific binding sites, the membranes were incubated overnight at 4°C with primary antibodies against human Pygo1 (Abcam, ab170607), c-Myc (Abcam, ab32072), survivin (Abcam, ab469), E-cadherin (Proteintech, 20874-1-AP), cleaved-caspase-3 (CST, #9664S), CyclinD1 (CST, #55506), RB (CST, #9313S), p16 (Proteintech, 10883-1-AP), p53 (Proteintech, 10442-1-AP), *β*-catenin (Proteintech, 17565-1-AP), p27Kip1 (Proteintech, 25614-1-AP), CyclinE1 (CST, #20808S), GAPDH (Proteintech, 10494-1-AP), and *β*-actin (Proteintech, 6008-1-Ig), respectively. The membranes were washed 3 times with TBST and incubated with a HRP-conjugated secondary antibody for 2 h at room temperature (Vazyme, China). After 3 washes with TBST, the membranes were finally incubated in Luminata Forte Western HRP Substrate (Millipore, USA) for 5 min and scanned using an Odyssey Infrared Imaging System (LI-COR Biosciences, USA). The labeled protein bands were quantified using ImageJ software (National Institutes of Health, USA), and the data were normalized to the expression levels of GAPDH proteins. The blots shown in the figures were representative of at least 3 experiments.

### 2.9. Coimmunoprecipitation (Co-IP) Assay

Protein solution was quantified using the Bradford method, and equal concentration was used from all samples. Cell extracts were first pretreated with 25 *μ*l of protein A/G-Magnetic beads (50% *v*/*v*, Bimake, China). The supernatants were immunoprecipitated with 5 *μ*g of anti-*β*-catenin antibodies overnight at 4°C, followed by incubation with protein A/G-agarose 4 h at 4°C. Negative control was immunoprecipitated with rabbit IgG or mouse IgG only. Protein A/G-Magnetic bead-antigen-antibody complexes were collected by centrifugation at 12,000 rpm for 60 s at 4°C. The pellets were washed 5 times with 1 ml of IPH buffer (50 mM Tris-HCl, pH 8.0, 150 mM NaCl, 5 mM EDTA, 0.5% Nonidet P-40, and 0.1 mM PMSF) for 5 min each time at 4°C. Bound proteins were resolved by SDS-PAGE, followed by western blotting with anti-*β*-catenin or anti-TCF4 (Abcam, ab76151) antibodies, respectively. The experiments were replicated ≥3 times.

### 2.10. In Vivo Xenograft Experiments

The mouse experiments were conducted in the animal facility of the College of Life Sciences, Hunan Normal University (Changsha, China), and approved by the Institutional Animal Care and Use Committee. Approximately 4-week-old male athymic Balb/c-nu/nu mice were purchased from Hunan SJA Laboratory Animal Co., Ltd. (Changsha, China). Mice were housed in a microventilation cage system (MVCS) with a computerized environmental control system (Threeshine Inc.). The temperature was maintained at 24°C with a relative humidity of 45-55%. The mice were provided a standard maintenance diet (Dae Han Bio Link). After acclimatization for 1 week, A549 cells stably transfected with control and Pygo1-encoding plasmids were trypsinized and resuspended in phosphate-buffered saline (pH 7.4). The cell suspensions were then mixed with Matrigel (vigorous; volume ratio, 1 : 1) at 4°C. The mixture containing 5 × 10^6^ cells in a volume of 100 *μ*l was s.c. injected into the flanks of male mice (3 mice/group). When the mean tumor size reached between 100 and 200 mm^3^, the mice were randomly divided into 2 groups (vehicle-treated and KYA1797K-treated groups; 3 male mice per group). KYA1797K (in a suspension of 90% PBS and 10% Tween 80) was injected i.p. 5 d per week at a dose of 20 mg/kg. Tumor volume and body weight were measured every 4 d. Tumors were measured using Vernier calipers, and tumor volume was calculated according to the following formula: *V* = 0.5(*L* × *W*^2^), where *L* is length and *W* is width. 40 days after drug treatment, the mice were killed, and the tumors were excised, weighed, and fixed in 4% PFA or snap-frozen in liquid nitrogen for further analysis.

### 2.11. Statistical Analysis

The data were presented as the means ± standard deviations (SD). GraphPad Prism 8.0 (GraphPad Software, USA) was used to conduct the statistical analysis. Student's *t*-test was used to compare the means of continuous variables between 2 groups. Statistical significance was indicated by ^∗^*p* < 0.05, ^∗∗^*p* < 0.01, and ^∗∗∗^*p* < 0.01. A *p* value of <0.05 was considered to indicate a significant difference.

## 3. Results

### 3.1. Pygo1 Expression Was Upregulated in Non-Small-Cell Lung Cancer

**Figure 1:**
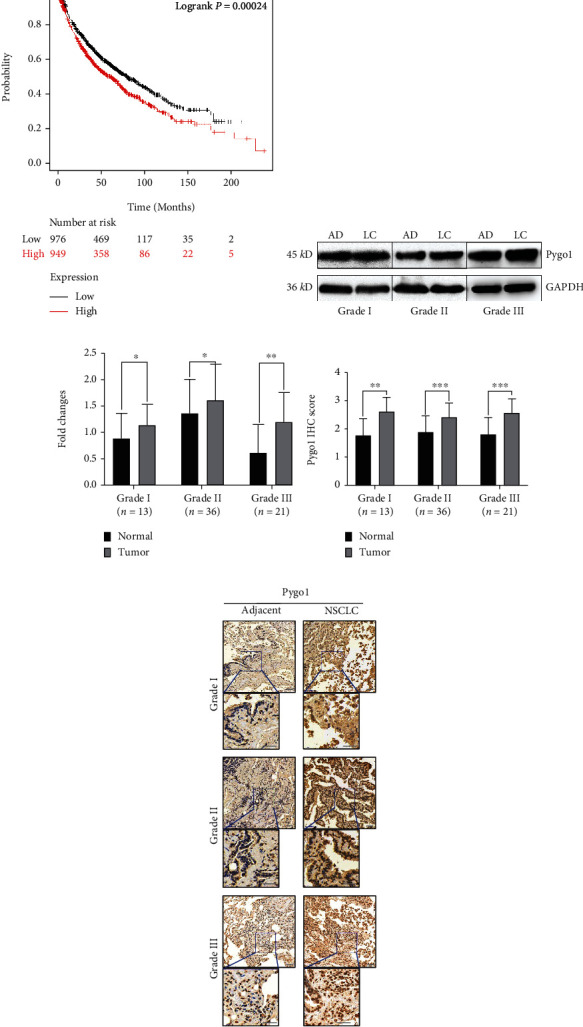
Pygo1 expression was upregulated in non-small-cell lung cancer. (a) Kaplan-Meier overall survival (OS) curves according to Pygo1 expression levels in database analyzed by Kaplan-Meier Plotter (nlow = 976, nhigh = 949). (b) Pygo1 protein levels in NSCLC tissues (LC) of different pathological grades and adjacent tissues (AD) were detected by western blotting. (c) Protein expression levels were quantified and analyzed by ImageJ and GraphPad Prism software, respectively. The results shown are representative of independent experiments. (d) Quantitative analysis of IHC images using ImageJ. (e) Representative images from immunohistochemistry (IHC) assays of NSCLC of different pathological grades and adjacent tissues. Grade I, grade II, and grade III refer to WHO-IA-LUAD, WHO-IIA-LUAD, and WHO-IIIB-LUSC, respectively. Scale bar: 100 *μ*m. ns *p* ≥ 0.05, ^∗^*p* < 0.05, ^∗∗^*p* < 0.01, and ^∗∗∗^*p* < 0.001.

To explore the clinical significance of Pygo1 in NSCLC, we analyzed the relationship between Pygo1 expression and overall survival (OS) using the Kaplan-Meier Plotter public database. A significant association of higher Pygo1 expression with reduced patient survival rates was observed (Figure 1(a)). Tissue samples collected from Hunan Cancer Hospital were introduced to conduct western blotting experiments, and the levels of Pygo1 protein in both cancer tissues and adjacent normal tissues were detected (Supplementary Materials: Figures S-1–S-3). As shown in Figures 1(b) and 1(c), Pygo1 protein was significantly upregulated in cancer tissues of different pathological grades (WHO-IA-LUAD, WHO-IIA-LUAD, and WHO-IIIB-LUSC) as compared with adjacent normal tissues. In addition, the immunohistochemical staining of cancer tissue sections further confirmed the increased expression of Pygo1 in NSCLC (Figures 1(d) and 1(e)). In summary, these data indicated that Pygo1 was upregulated in NSCLC.

### 3.2. Pygo1 Overexpression In Vitro Regulated Tumorigenesis by a *β*-Catenin-Dependent Mechanism

**Figure 2:**
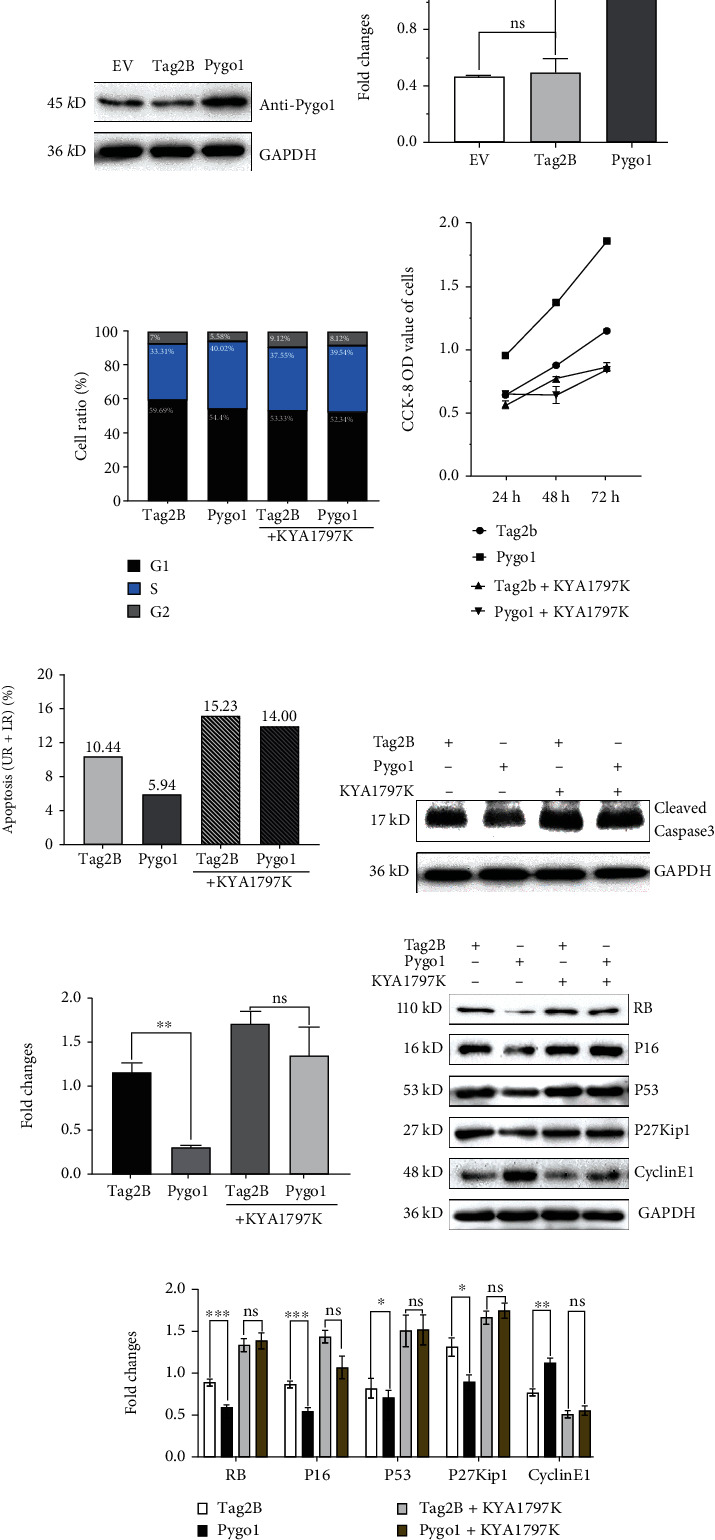
Overexpression of Pygo1 induced G1/S phase transformation of A549 cells and inhibited apoptosis. (a, b) Pygo1 protein was overexpressed in A549 cell lines and its fold change. EV: blank control; Tag2B: negative control; Pygo1: Pygo1 overexpression cells; GAPDH: loading control (*n* = 3). (c) Quantitative analysis of cell cycle stage by flow cytometry. (d) Quantitative analysis of cell proliferation detected by CCK-8. (e) Quantitative analysis of apoptosis by flow cytometry. (f, g) Cleaved-caspase-3 protein level detected by western blotting and its fold change. (h, i) Cell cycle-related proteins detected by western blotting and their fold change by ImageJ and GraphPad Prism (*n* = 3). ns *p* ≥ 0.05, ^∗^*p* < 0.05, ^∗∗^*p* < 0.01, and ^∗∗∗^*p* < 0.001.

To determine the role of Pygo1 in the tumorigenesis of NSCLC, we next subjected A549 cells to transient transfection with a Pygo1 overexpression vector or empty Tag2B vector (control) (Figures 2(a) and 2(b)). The proportion of cells in the S phase exhibited a 6.71% increase in the Pygo1-overexpressing cells through a flow cytometry analysis, whereas only a 1.99% increase was observed in the KYA1797K-treated cells (Figure 2(c)). Results of western blotting in Pygo1-overexpressing cells revealed significant downregulation of a series of cell cycle-related proteins, including RB, p16, p53, and p27Kip1, accompanied by the upregulation of CyclinE1. Again, these Pygo1-induced changes were abolished by KYA1797K treatment (Figures 2(h) and 2(i)).

An *in vitro* CCK-8 assay demonstrated that Pygo1 overexpression caused a significant increase of cell proliferation (Figure 2(d)). However, such increase was completely abolished by KYA1797K treatment. While a reduced expression of caspase-3 protein was examined in Pygo1 overexpressed cells, the reduction was significantly inhibited by KYA1797K treatment, indicating an inhibiting role of Pygo1 in cell apoptosis (Figures 2(e)–2(g)). In summary, our data suggested that Pygo1 induced G1/S cycle transformation, promoted cell proliferation, and inhibited apoptosis at least partly through *β*-catenin-dependent mechanism.

### 3.3. Pygo1 Overexpression Enhanced A549 Cell Invasion, Migration Ability, and Colony Formation In Vitro

**Figure 3:**
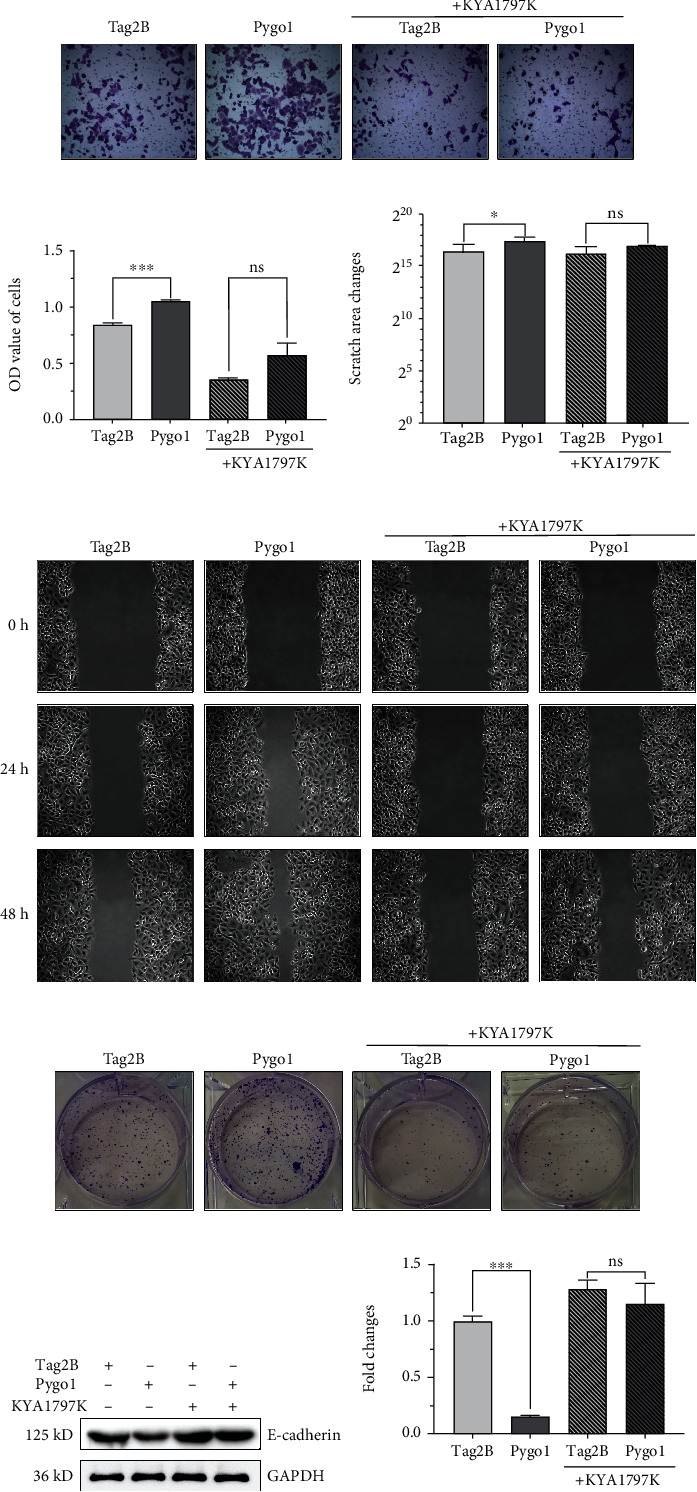
Pygo1 overexpression in vitro enhanced A549 cell invasion and migration ability and promoted colony formation. (a, b) Transwell experiments and repeated measurement of absorbance (OD) values. (c, d) Wound healing test result visualization and repeated measurement of scratch area changes at different time points. (e) Visualization of colony formation assay results. (f, g) Western blotting analysis of cell lysates with different treatments using rabbit polyclonal antibody to E-cadherin. Quantitative analysis of protein expression levels using ImageJ and GraphPad Prism (*n* = 3). ns *p* ≥ 0.05, ^∗^*p* < 0.05, ^∗∗^*p* < 0.01, and ^∗∗∗^*p* < 0.001.

Subsequently, the Transwell invasion assay was performed to determine the effect of Pygo1 on invasiveness of A549 cells. It can be seen from Figures 3(a) and 3(b) that evidently enhanced cell invasion by Pygo1 overexpression was significantly attenuated by KYA1797K treatment. Pygo1 overexpression also significantly enhanced the migration abilities of A549 cells. However, no significant difference was observed between the Tag2B control and Pygo1 overexpression groups subjected to KYA1797K treatment (Figures 3(c) and 3(d)). Furthermore, Pygo1 overexpression significantly downregulated the expression of E-cadherin protein, which was restored by KYA1797K treatment (Figures 3(f) and 3(g)). Our clone formation experiments uncovered that an obvious increase of colony formation induced by Pygo1 overexpression was effectively reversed by KYA1797K treatment (Figure 3(e)). In summary, these data indicated that Pygo1 enhanced the invasiveness, migration ability, and colony formation ability of A549 cells through a *β*-catenin-dependent mechanism.

### 3.4. Pygo1 Promoted the Tumorigenesis of NSCLC in a Canonical Wnt Signaling Pathway-Dependent Mechanism

**Figure 4:**
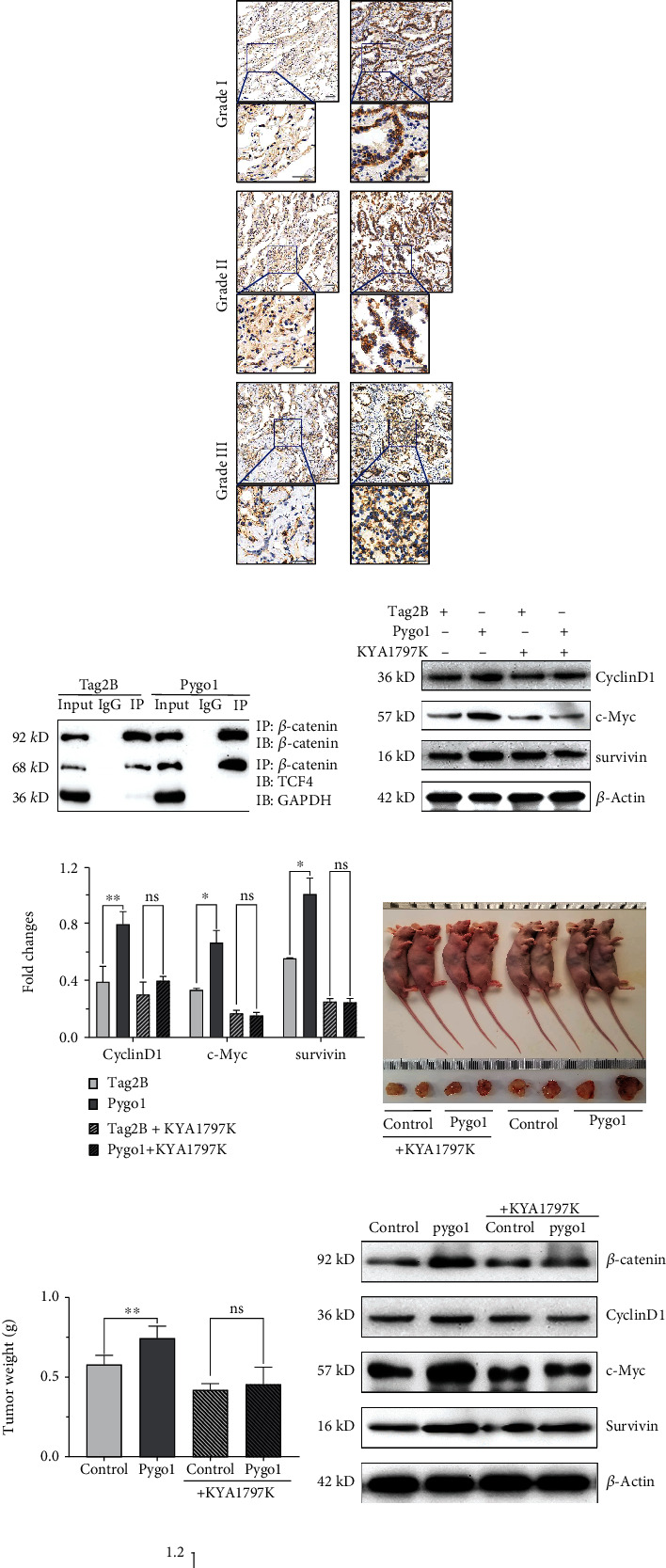
Pygo1 upregulation in NSCLC was related to the Wnt signaling pathway. (a, b) Western blotting analysis of lysates with different treatments using rabbit polyclonal antibody to *β*-catenin, GAPDH was used as a loading control (*n* = 3). (c) Representative images of immunohistochemical (IHC) analysis of *β*-catenin expression levels in NSCLC from different pathological grades and adjacent tissues and (d) quantitative analysis of IHC images using ImageJ. Grade I, grade II, and grade III refer to WHO-IA-LUAD, WHO-IIA-LUAD, and WHO-IIIB-LUSC, respectively. Scale bar: 100 *μ*m. ns *p* ≥ 0.05, ^∗^*p* < 0.05, ^∗∗^*p* < 0.01, and ^∗∗∗^*p* < 0.001. (e) Coimmunoprecipitation analysis of TCF4 and *β*-catenin expression in A549 cells. (f, g) Western blotting analysis of Wnt/*β*-catenin signal proteins and quantitative analysis (*n* = 3); ns *p* ≥ 0.05, ^∗^*p* < 0.05, ^∗∗^*p* < 0.01, and ^∗∗∗^*p* < 0.001. (h) Necropsy photographs of mice and xenograft tumors on day 41. The results shown are representative of independent experiments. (i) Tumor weight was measured on day 41 of the experiment (mean ± SE). (j, k) Western blot analysis of the tissues samples and quantitative analysis (*n* = 3); ns *p* ≥ 0.05, ^∗^*p* < 0.05, ^∗∗^*p* < 0.01, and ^∗∗∗^*p* < 0.001. Equal amounts of lysates were analyzed via western blot analysis and then probed for different proteins.

To investigate whether Pygo1 regulates Wnt signaling pathway transcriptional activity in lung cancer cells, we then evaluated the levels of *β*-catenin in Pygo1 overexpressed A549 cells. Western blotting analysis indicated increased protein levels of *β*-catenin followed by Pygo1 overexpression, although this trend was alleviated by KYA1977K treatment (Figures 4(a) and 4(b)). Immunohistochemical analysis was consistent with the *in vitro* results; that is, high *β*-catenin signal in cancer tissues was accompanied with high expressing levels of Pygo1 (Figures 4(c) and 4(d)). Co-IP assay revealed that Pygo1 enhanced *β*-catenin and TCF4 interaction (Figure 4(e)); Pygo1 overexpressed cells also induced higher expression levels of Wnt-targeted genes, such as c-Myc, CyclinD1, and survivin (Figures 4(f) and 4(g)). Moreover, the xenograft model experiments demonstrated that overexpression of Pygo1 promoted tumor formation (Figures 4(h) and 4(i)). Western blotting results of tumor tissues were in agreement with the *in vitro* experiments (Figures 4(j) and 4(k)).

## 4. Discussion

Abnormal activation of the classical Wnt signaling pathway plays an important role in various types of cancer. Pygo and related protein family members, the Wnt pathway component, have previously been demonstrated to be involved in *β*-catenin/TCF-mediated colorectal and breast cancer cell proliferation [11, 18]. Pygo2 might play an important role in lung cancer [14]. In the present study, we reported that Pygo1 was upregulated in pathological samples of human NSCLC. The overexpression of Pygo1 in lung cancer cells *in vitro* promoted cancer cell proliferation and migration, but inhibited cell apoptosis. Increased nuclear levels of *β*-catenin were observed simultaneously, while the inhibition of *β*-catenin appeared to partially block the effects of Pygo1 overexpression. Our current observations suggested that Pygo1 regulated the behavior of human NSCLC through the canonical Wnt/*β*-catenin signaling pathway.

### 4.1. Pygo1 Promotes Lung Cancer

Here, we demonstrated that Pygo1 was upregulated in NSCLC tissues collected from patients with early- or middle-stage disease. The consistent upregulated expression of Pygo1 in LUAD I, LUAD II, and LUSC III tissue samples suggested that Pygo1 might be closely related to the occurrence of lung cancer. Bioinformatics prediction also showed the prognosis of lung tumors with high expression of Pygo1. Moreover, *in vitro* data showed that Pygo1 promoted lung cancer, induced cell cycle transformation, and inhibited apoptosis, which led to an expansion of the cell population. In addition, our *in vitro* data also showed that Pygo1 promoted lung cancer cell clone formation and migration, suggesting that this protein might also regulate tumor metastasis. Taken together, our results demonstrated that Pygo1 exerted a wide range of cell biological functions in tumorigenesis and tumor development.

### 4.2. Pygo1 Promotes Lung Cancer through a Mechanism Dependent on Canonical *β*-Catenin Signaling

Previous studies have revealed abnormal activation of the classic Wnt signaling pathway in lung cancers [19]. Wnt/*β*-catenin signaling has been shown to regulate cancer by controlling the cell cycle and apoptosis [20]. Specifically, activation of this pathway suppresses the degradation of *β*-catenin, thus enabling an interaction between free *β*-catenin and TCF that promotes the transcriptional activation of target genes encoding antiapoptotic factors such as c-Myc, CyclinD1, and survivin [21]. In contrast, treatment with monoclonal antibodies or RNA interference (RNAi) specific for Wnt-1/Wnt-2 signaling induced apoptosis [22]. Pygo has been proven to be the core factor that interacts with *β*-catenin in various model organisms. Our current findings suggested that Pygo1 overexpression induced activation of Wnt/*β*-catenin signaling and increased expression of the related target genes encoding c-Myc, CyclinD1, and survivin, both *in vitro* and *in vivo*. Attenuation of Pygo1-induced changes via employment of KYA1797K, which blocked Wnt/*β*-catenin signaling, was in line with previous studies [23, 24]. KYA1797K also eliminated the effects of Pygo1 overexpression on lung cancer cell proliferation, apoptosis, colony formation, and tumor formation inside the body, suggesting that Pygo1 played an extensive and canonical Wnt pathway-dependent role in lung cancer.

c-Myc is a well-known oncogene, whose upregulation contributes to the spontaneous proliferation and growth of cancer cells [25, 26]. CyclinD1 can regulate the transition of G1 and S phase, which is closely related to the proliferation of cancer cells [27, 28]. Survivin, a product of a Wnt signaling downstream gene, may participate indirectly in the inactivation of caspase-3 through its interaction with CDK4 or with the procaspase-3/p21 complex [29] to accelerate cell proliferation and inhibit apoptosis. In agreement with a previous study which reported that caspase-3 was the main caspase in *β*-catenin cleavage *in vivo* [30], we observed that A549 cell apoptosis in response to Pygo1 inhibition was accompanied by the downregulation of caspase-3. Furthermore, the inhibition of Wnt signaling restored the expression of caspase-3 in A549 cells.

Our current observations also suggested that Wnt/*β*-catenin partially mediated the migration of Pygo1 to lung cancer cells. Decreased expression of E-cadherin in the cell membrane, loss of cell-to-cell adhesion, and EMT of cell morphology are the initiating factors for cancer cells to acquire the ability to migrate [31, 32]. Studies have shown that Wnt/*β*-catenin signaling could upregulate the expression of Slug (Snai2), ZEB1, and ZEB2 in cancer cells, thereby reducing the level of E-cadherin to promote cell migration [33, 34]. These speculations reasonably explained the results of our cell invasion and wound healing experiments, and the change in the expression level of E-cadherin was also consistent with the trend of the above results. Potentially, in our experiments, Pygo1 overexpression might have disrupted the connections between E-cadherin and *β*-catenin molecules, thus enabling an increase in the nuclear level of *β*-catenin and further promotion of cell migration.

Interestingly, Pygo1 promotes cancer through Wnt/*β*-catenin signaling, which seems to occur in human lung cancer patients. Consistent increased *β*-catenin nuclear signaling was observed in human Pygo1 highly expressed NSCLC tissues. Moreover, *in vitro* experimental data showed that overexpression of Pygo1 in lung cancer cells could promote *β*-catenin/TCF4 complex formation. The mechanism of these changes might have two reasons. One was that the high expression of Pygo1 enhances the affinity of *β*-catenin and TCF4 [35]; the other was that Pygo1 directly upregulated the expression of *β*-catenin. In our data, overexpression of Pygo1 did upregulate *β*-catenin expression, which might be a positive feedback mechanism in the Wnt/*β*-catenin signaling pathway, because our data also showed that *β*-catenin expression was slightly downregulated after KYA1797K treatment. It appears to be a positive feedback mechanism in the Wnt/*β*-catenin signaling pathway, which is coincident with a recent publication [36].

In any case, it is understandable that the overexpression of Pygo1 in patient tissue cells leads to the accumulation of *β*-catenin signals in the nucleus [37]. Our results supported a positive correlation between Pygo1 expression and the nuclear *β*-catenin level in lung cancer patients, indicating that Pygo1 induced lung cancer through a *β*-catenin-dependent mechanism.

We noted that in our study overexpression of Pygo1 was accompanied by a decrease of P53 levels. Previous studies have shown that there is a regulatory relationship between P53 and the Wnt signaling pathway, but it is unclear whether the same situation exists in this study [38]. The mechanism underlying this association remains unclear. Previous studies have demonstrated the complexity of Wnt signaling and observed the potential interactions between Wnt signaling and other pathways as for enhancing or inhibiting tumor aggressiveness [39], which is a topic that warrants further exploration in future studies.

## Data Availability

All the data used to support the findings of this study are included within the article.

## Supplementary Materials

Supplementary Materials In the content of the supplementary material, the manuscript western blot raw data (Figure S-1 to Figure S-7) and no display of results (Figure S-8) are added in the manuscript.
