# Supplementary material for: Pygo1 Regulates the Behavior of Human Non-Small-Cell Lung Cancer via the Wnt/β-Catenin Pathway
Source: Dis Markers. 2022 Nov 8;2022:6993994. doi: 10.1155/2022/6993994 (PMC9666017; doi:10.1155/2022/6993994)

**Supplementary materials**

**Figure legend**

**Figure S-1.** Pygo1 protein levels in NSCLC tissues (LC) of different pathological grades and adjacent tissues (AD) were detected by western blotting. Visual map of western blot results of 13 cases of Grade I; Grade I are refered to WHO-IA-LUAD.

**Figure S-2.** Pygo1 protein levels in NSCLC tissues (LC) of different pathological grades and adjacent tissues (AD) were detected by western blotting. Visual map of western blot results of 36 cases of Grade II; Grade II are refered to WHO-IIA-LUAD.

**Figure S-3.** Pygo1 protein levels in NSCLC tissues (LC) of different pathological grades and adjacent tissues (AD) were detected by western blotting.Visual map of western blot results of 21 cases of Grade III; Grade III are refered to WHO-IIIB-LUSC.

**Figure S-4.** (A)Pygo1 protein was overexpressed in A549 cell lines. EV, blank control; Tag2B, negative control; Pygo1, Pygo1 overexpression cells; (B) Cleaved-Caspase3 protein level detected by western blotting and its results of another repeated independent experiment.

**Figure S-5.** Western blot analysis the A549. (A) RB protein level detected by western blotting; (B) P16 protein level detected by western blotting; (C) P53 protein level detected by western blotting; (D) P27Kip1 protein level detected by western blotting; (E) Cyclin E1 protein level detected by western blotting; (F) Internal reference control of GAPDH corresponding to RB, P16, P53, E-cadherin；(G) E-cadherin protein level detected by western blotting.

**Figure S-6.** Western blot analysis the A549. (A) *β*-catenin protein level detected by western blottingt; (B) Cyclin D1 protein level detected by western blotting; (C) c-Myc protein level detected by western blotting; (D) survivin protein level detected by western blotting; (E) Internal reference control of *β*-actin corresponding to *β*-catenin, Cyclin D1, c-Myc, surviving.

**Figure S-7.** Western blot analysis the tissues samples (A) *β*-catenin protein level detected by western blotting; (B) Cyclin D1 protein level detected by western blotting; (C) c-Myc protein level detected by western blotting; (D) survivin protein level detected by western blotting; (E) Internal reference control of *β*-actin corresponding to *β*-catenin, Cyclin D1, c-Myc, surviving. Control, negative control; Pygo1, Pygo1 overexpression.

**Figure S-8.** (A) The colony formation assay; (B) The tumor volume measured data as tumor growth.


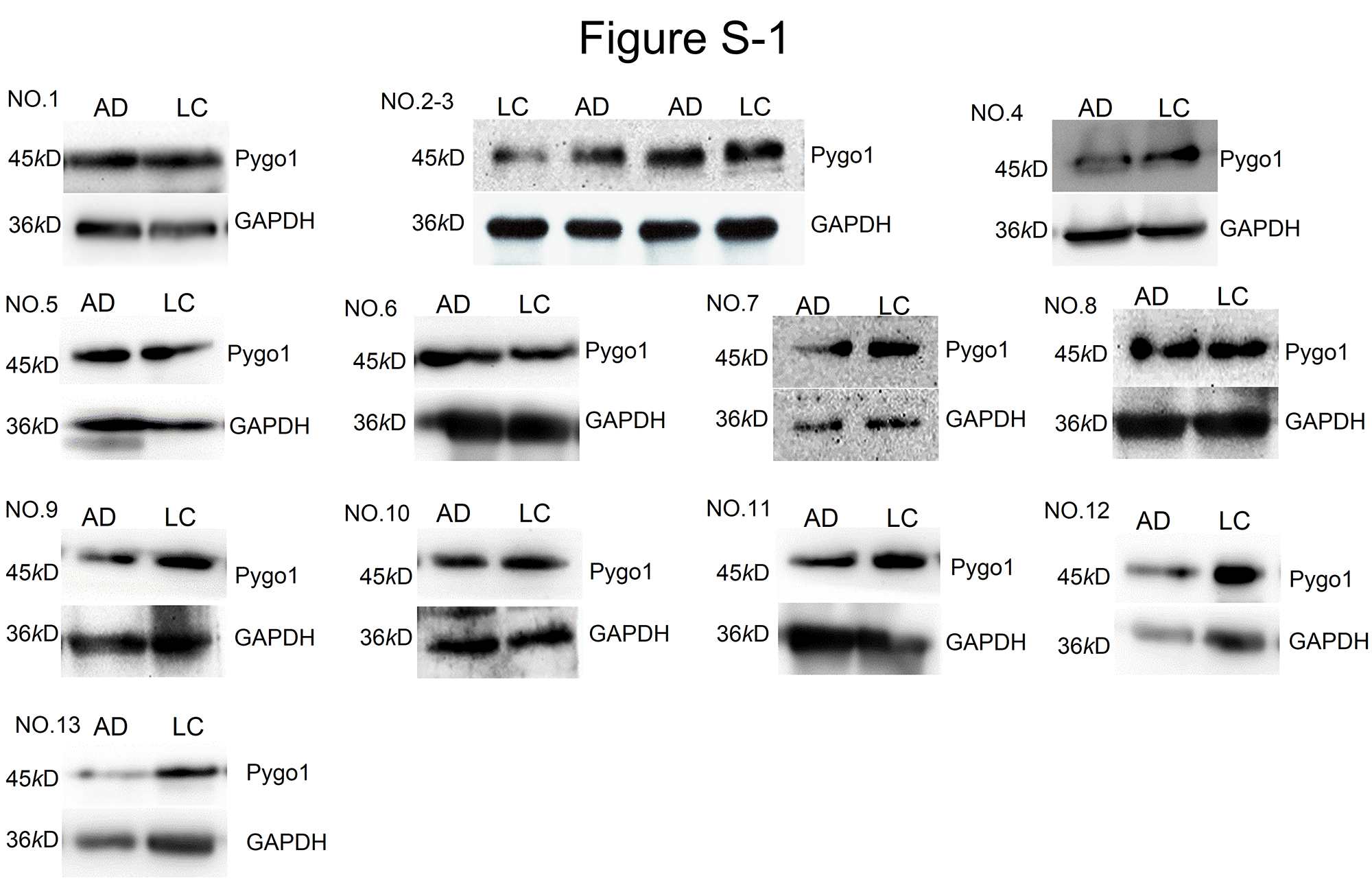

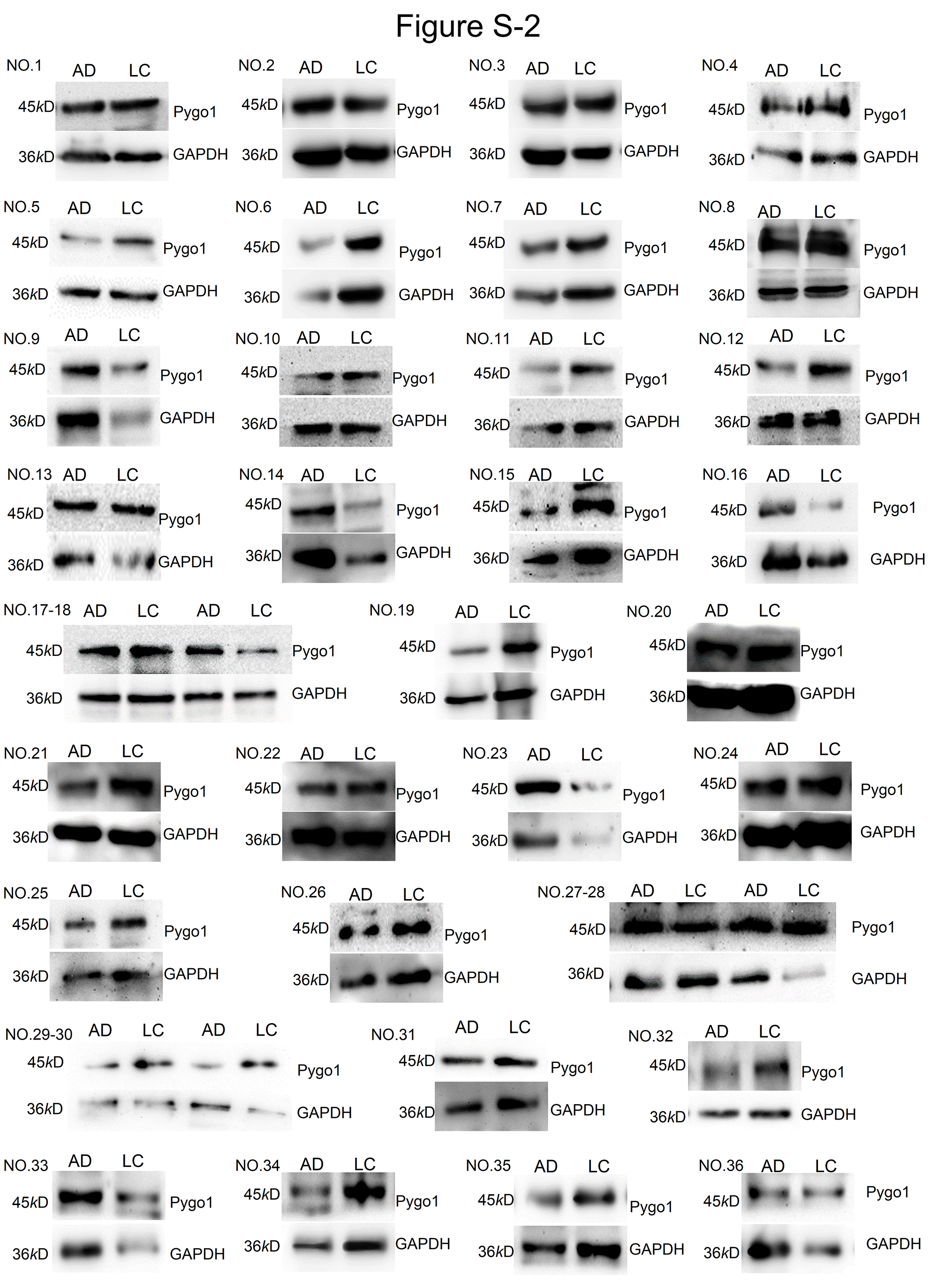

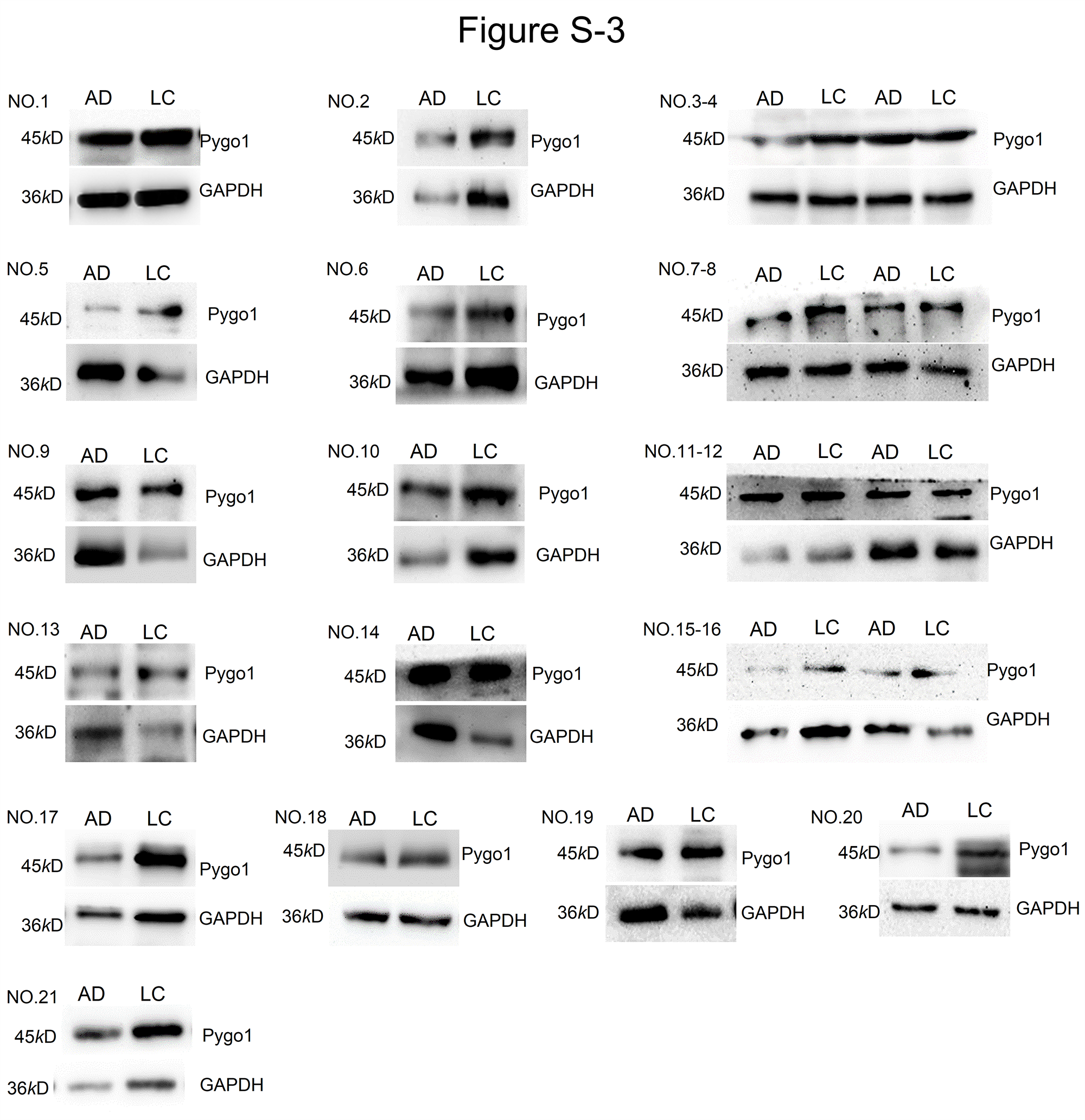

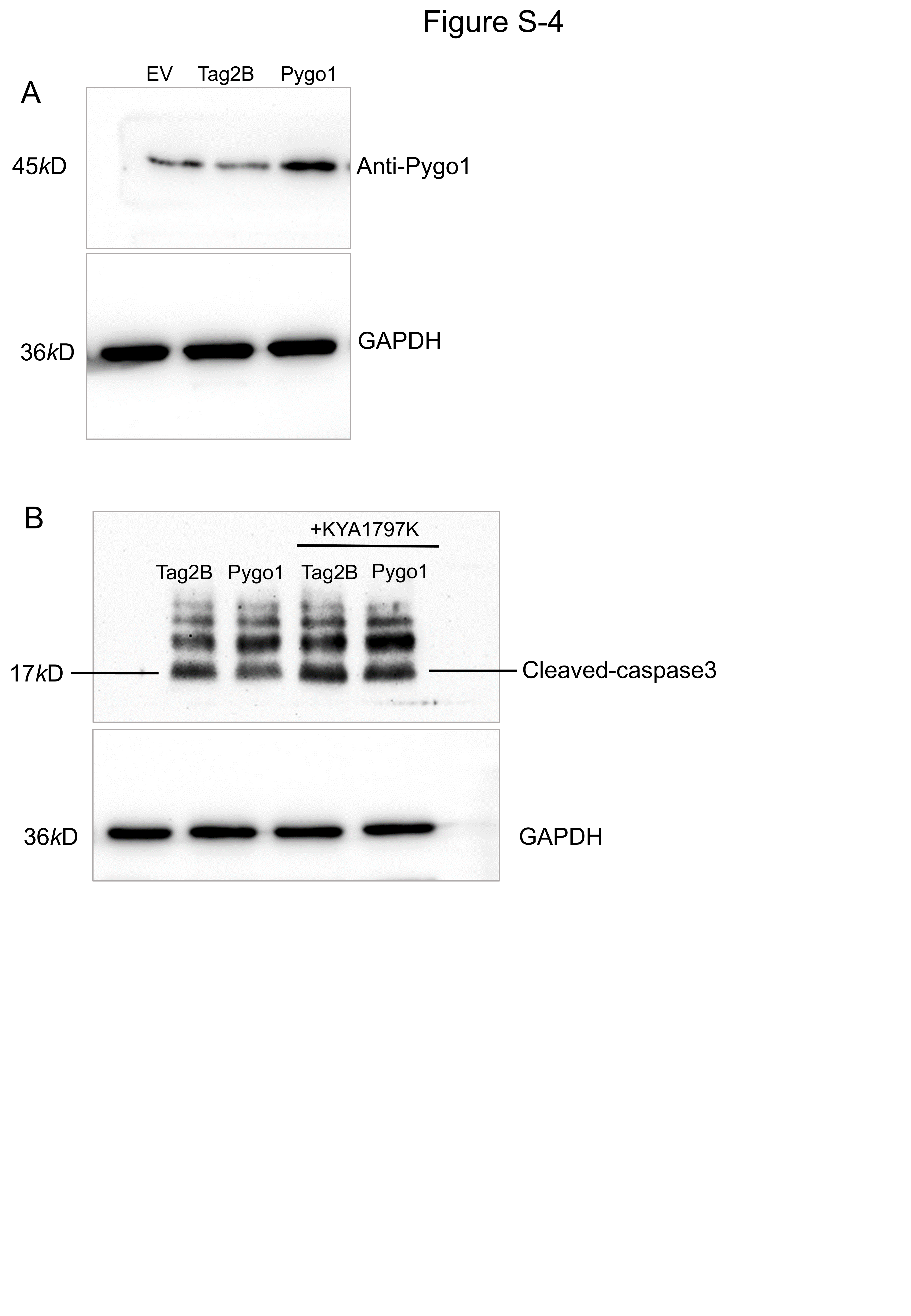

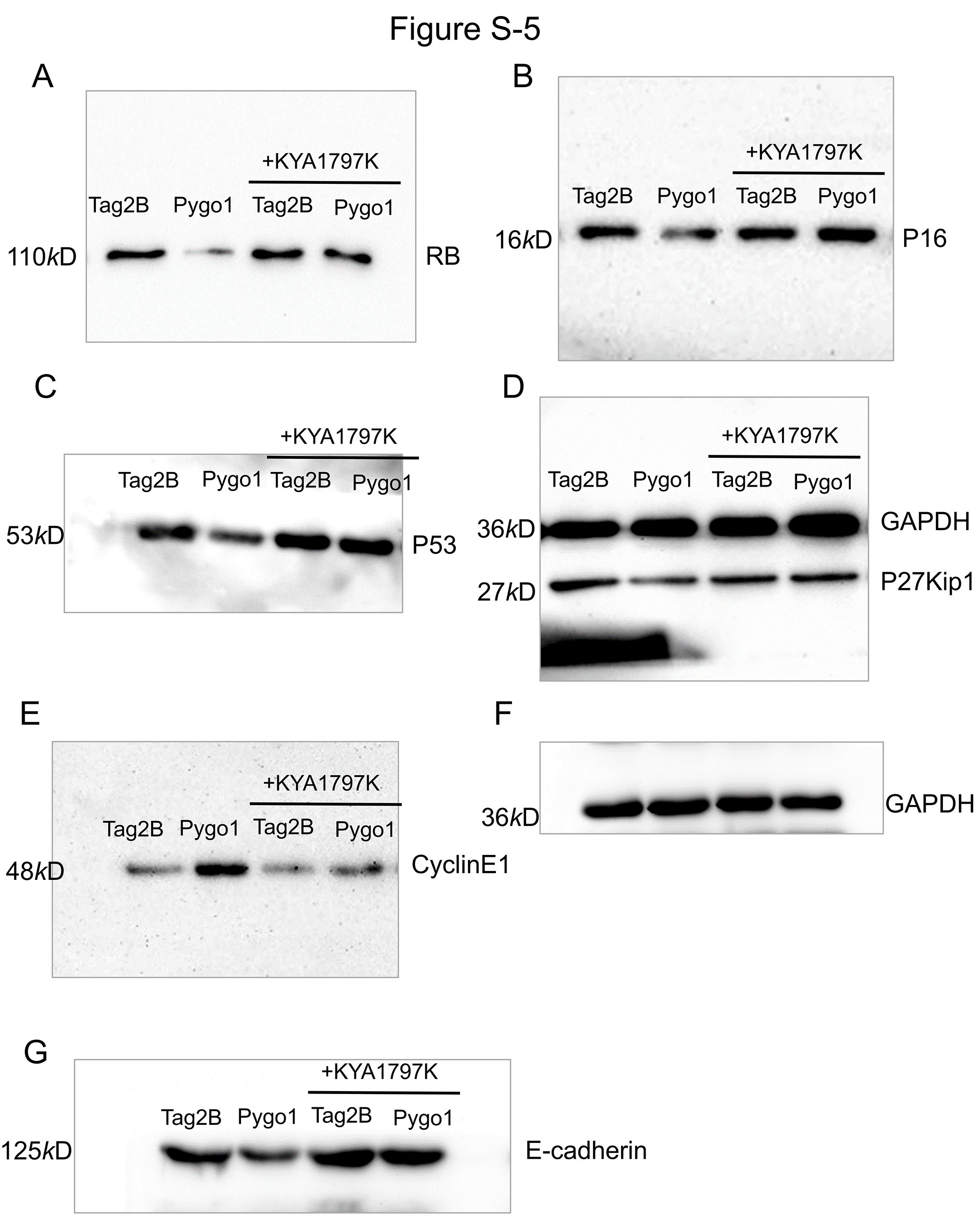

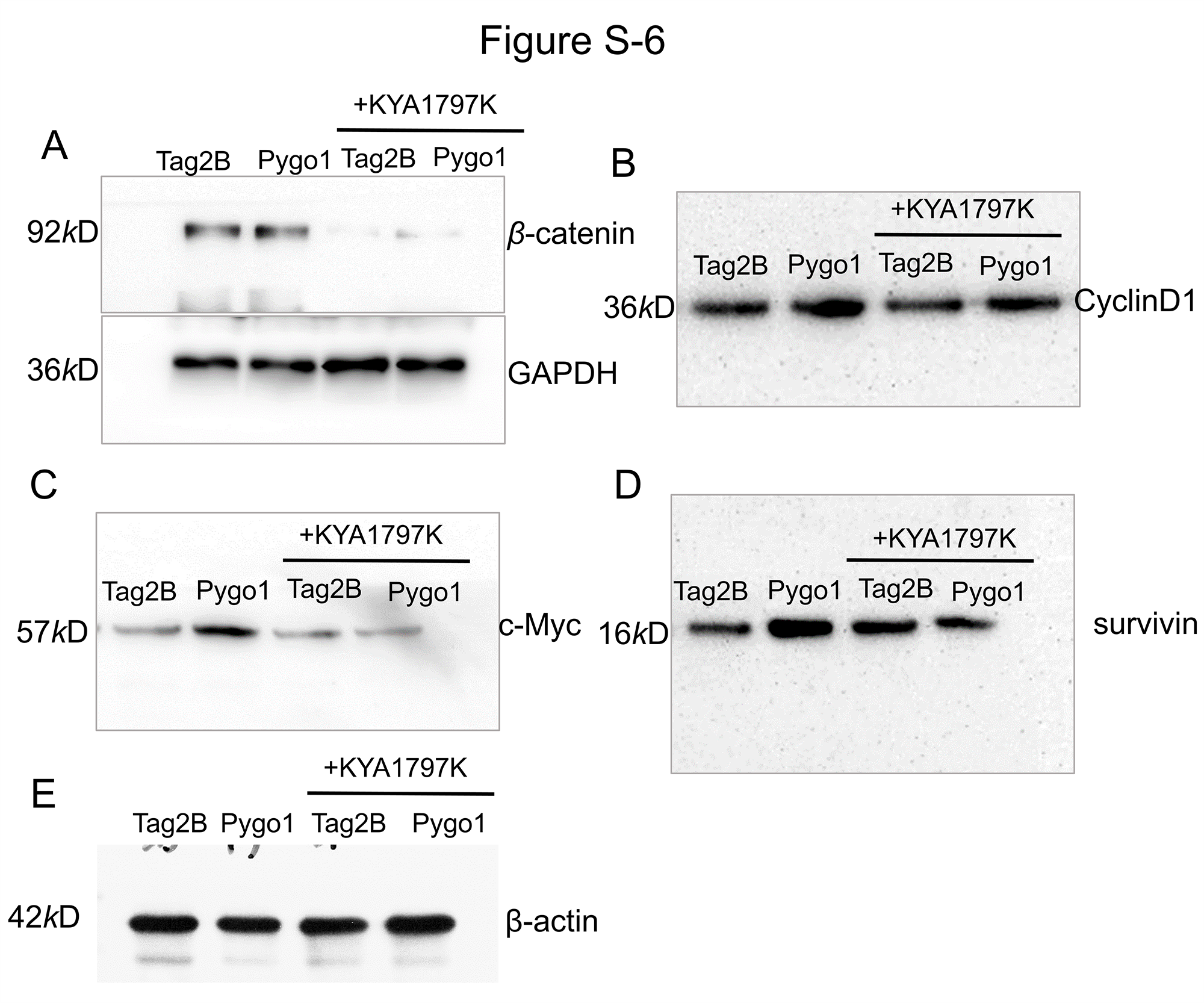

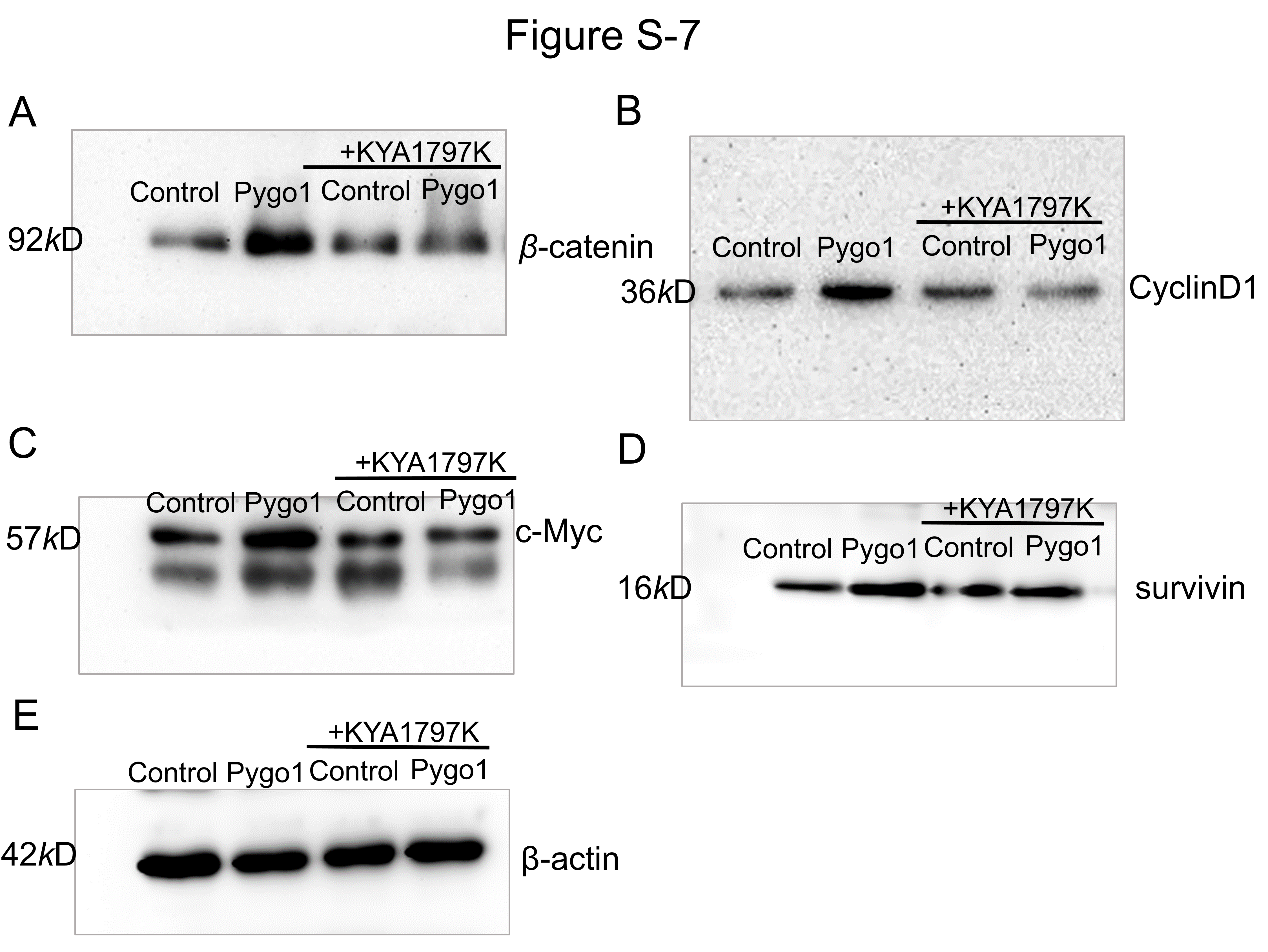


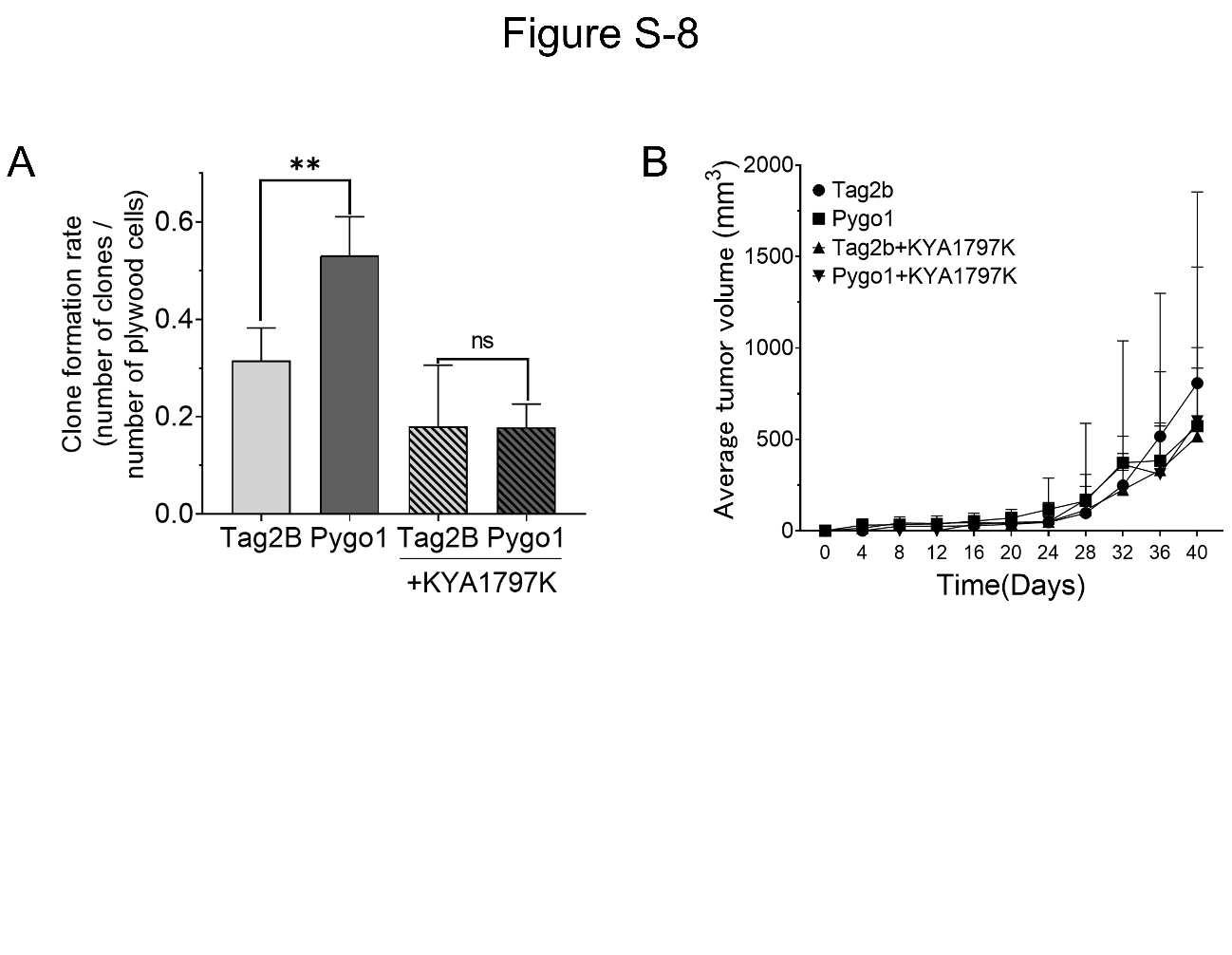

Supplement: Supplementary Materials — In the content of the supplementary material, the manuscript western blot raw data (Figure S-1 to Figure S-7) and no display of results (Figure S-8) are added in the manuscript. [file 6993994.f1.docx]
